# Understanding the effect of gender-based violence on uptake and utilisation of HIV prevention, treatment, and care services among transgender women: a qualitative study in the greater Kampala metropolitan area, Uganda

**DOI:** 10.1186/s12905-023-02402-3

**Published:** 2023-05-09

**Authors:** Naume Muyanga, John Bosco Isunju, Tonny Ssekamatte, Aisha Nalugya, Patience Oputan, Juliet Kiguli, Simon Peter S. Kibira, Solomon Tsebeni Wafula, David Ssekamatte, Richard K. Mugambe, Rhoda K. Wanyenze, Luisa Orza

**Affiliations:** 1 Programs Department, Transgender Equality Uganda, Kampala, Uganda; 2 Department of Disease Control and Environmental Health, Makerere University School of Public Health, New Mulago Hill Road, Mulago Kampala, Uganda; 3 Department of Community Health and Behavioural Sciences, Makerere University School of Public Health, New Mulago Hill Road, Mulago Kampala, Uganda; 4 Department of Management, Uganda Management Institute, K.A.R. Road, Kampala, Uganda; 5 Frontline AIDS, 35 New England Street, Brighton, United Kingdom

**Keywords:** Transwomen, Gender-based violence, HIV/AIDS, Uganda, Stigma

## Abstract

**Background:**

Transwomen (also known as transgender women) are disproportionately affected by all forms of gender-based violence (GBV). The high prevalence of physical, sexual and emotional violence not only predisposes transwomen to HIV infection but also limits the uptake/access to HIV prevention, care, and treatment services. Despite the high prevalence of HIV infection and GBV among transwomen, there is limited evidence on how GBV affects the uptake and utilisation of HIV prevention, care, and treatment services. Therefore, this qualitative study explored how GBV affects uptake and utilisation of HIV prevention, treatment, and care services among transwomen in the Greater Kampala Metropolitan Area (GKMA), Uganda.

**Methods:**

This participatory qualitative study was conducted among transwomen in the GKMA. A total of 20 in-depth interviews, 6 focus group discussions, and 10 key informant interviews were conducted to explore how GBV affects the uptake and utilisation of HIV prevention, treatment, and care services among transwomen. Data were analysed using a thematic content analysis framework. Data were transcribed verbatim, and NVivo version 12 was used for coding.

**Results:**

At the individual level, emotional violence suffered by transwomen led to fear of disclosing their HIV status and other health conditions to intimate partners and healthcare providers respectively; inability to negotiate condom use; and non-adherence to antiretroviral therapy (ART). Sexual violence compromised the ability of transwomen to negotiate condom use with intimate partners, clients, and employers. Physical and emotional violence at the community level led to fear among transwomen traveling to healthcare facilities. Emotional violence suffered by transwomen in healthcare settings led to the limited use of pre-exposure prophylaxis and HIV testing services, denial of healthcare services, and delays in receiving appropriate care. The fear of emotional violence also made it difficult for transwomen to approach healthcare providers. Fear of physical violence such as being beaten while in healthcare settings made transwomen shun healthcare facilities.

**Conclusion:**

The effects of GBV on the uptake and utilisation of HIV prevention, care, and treatment services were observed in individual, community, and healthcare settings. Across all levels, physical, emotional, and sexual violence suffered by transwomen led to the shunning of healthcare facilities, denial of healthcare services, delays in receiving appropriate care, and the low utilisation of post-exposure prophylaxis, and HIV testing services. Given its effects on HIV transmission, there is a need to develop and implement strategies/ interventions targeting a reduction in GBV. Interventions should include strategies to sensitize communities to accept transwomen. Healthcare settings should provide an enabling environment for transwomen to approach any healthcare provider of their choice without fear of experiencing GBV.

## Background

Transwomen, defined as women who were assigned the male sex at birth, are still disproportionately affected by gender-based violence (GBV) and HIV in various regions of the world [1–5]. Globally, it is estimated that more than 19% of transwomen live with HIV [6]. The risk of HIV infection is 34 and 14 times greater among transwomen than other adults and adult women respectively [7, 8]. Furthermore, viral load suppression and retention in care are lower among transwomen than their non-transgender counterparts [9] [5–7]. There is limited data on the epidemiology of HIV infection among transgender populations. However, available estimates indicate a prevalence as high as 28.4% in Eastern and Southern Africa [8]. A prevalence of 20% has been reported among transwomen in Uganda [10]. An increased risk of death due to HIV-related causes among transwomen has also been reported in literature [11]. The high prevalence of HIV infection among transwomen is associated with GBV[12], which limits access to HIV prevention, care, and treatment services. Due to the low self-esteem and disempowerment transwomen are also more likely to use psychoactive substances and inconsistently use condoms [10, 13], which increases their vulnerability to HIV infection [10, 12, 14].

Data on GBV especially in low and middle-income countries are limited. Recent evidence however, indicates that GBV among transwomen is highly prevalent, frequent, often severe, and gravely under-reported [1, 15, 16]. GBV refers to violence perpetrated against an individual based on their gender or gender identity and can be physical, sexual, economic, emotional/ psychological in nature [1, 17]. Physical violence includes the intentional use of physical force with the potential to cause death, disability, injury, or harm [18, 19]. Examples of physical violence include punching, kicking, whipping, beating with an object, strangling, suffocating, attempted drowning, burning intentionally, and using or threatening with a weapon. Sexual violence includes abusive sexual touching, attempted forced or pressured sex, non-consensual vaginal, anal, or oral sex, and physically forced sex [18, 19]. Emotional violence (also known as psychological violence) refers to a pattern of behavior characterized by insults, humiliation, and instillation of fear in an individual to control them. Such acts include telling someone that they are not loved, wishing that they were dead or not born, and ridiculing someone [18, 19]. Economic violence includes taking away someone’s earnings, denying job opportunities due to someone’s gender identity, or making the victim unfit for work through targeted physical abuse [19, 20].

GBV among transwomen is often perpetrated by a variety of actors, ranging from intimate partners, friends, family, and community members, to representatives of the state (e.g., law enforcement officials and health care providers), on the basis of stigmatization of gender non-conformity, gender expression or identity, and perceived sexual orientation [1, 12, 16, 21]. Furthermore, transwomen’s economic vulnerabilities, including dependence on their intimate partners and engagement in sex work for survival amidst economic discrimination make them more vulnerable to GBV [22, 23]. In addition to other negative consequences [24], GBV has been shown to increase survivors’ vulnerability to HIV through intermediate-risk factors including coerced sex, multiple sex partners, unprotected sex, substance use, poor access to healthcare services, limited access to justice, negative mental health, emotional repercussions such as suicidal behaviors, depression, and social isolation [1, 5, 14, 25, 26]. Although a link between GBV and HIV infection has been established [17, 27], there is limited evidence on how GBV affects the uptake of HIV-related services among transwomen.

A review of the literature showed that most estimates of GBV are focused only on the experiences of cisgender, heterosexually identified women, with limited documented evidence among the transwomen sub-population [21]. In addition, the effect of GBV on the HIV continuum of care, from HIV prevention to HIV treatment and care is unclear.

Therefore, this study used the socio-ecological model to explore how GBV at multiple levels (i.e. individual, community and healthcare facility levels) affects the uptake and utilisation of HIV prevention, treatment and care services among transwomen in the greater Kampala Metropolitan area (GKMA), Uganda. The socio-ecological model can be used to understand the complex relationships among individuals, relationships, communities, and societal factors and how they affect the utilisation of prevention services [28–30]. Individual factors include one’s demographics, attitudes, beliefs and behaviours; relationships include the influence of friends and social networks on one’s behaviour; the community includes settings such as healthcare facilities, where social relationships occur or are constructed; and society includes social and cultural norms, and policies that create a climate for violence to occur [28]. The socio-ecological model has previously been used to understand help-seeking following exposure to GBV among transwomen in GKMA [16], and elsewhere to understand GBV experiences of the transgender populations [30–33]. Our findings can be used by policy makers to design interventions targeted at reducing the impact of GBV on the uptake and utilisation of HIV prevention, treatment and care services across socio-ecological scales.

## Materials and methods

### Study design and setting

A participatory qualitative approach of data collection was used. A narrative qualitative inquiry was used, the details of which are provided in our earlier publication [16]. This study was conducted in GKMA. GKMA comprises of Kampala, Mukono and Wakiso districts, and covers an area of 970 km2. It is the major business and industrial hub of Uganda contributing 70 per cent of the country’s industrial production and over 60 per cent of the country’s GDP. The Greater Kampala is the most populated region in Uganda. With about 2.7 million residents, Wakiso district has the highest population, followed by Kampala district with approximately 1.7 million, while Mukono district is the 7th most populated district in Uganda, with 701,400 people [34]. The GKMA is home to four (Mulago, Kiruddu, Kawempe and Butabika) national referral hospitals, one (Mulago) of which hosts the Most At Risk Population Initiative (MARPI), a key population-friendly friendly clinic. GKMA is also home to the majority of Uganda’s transgender-friendly healthcare facilities and civil society organisations.

### Study population

The study population included transwomen residing in the GKMA. Transwomen in the current study, were defined as women aged 18 years and above, who were assigned male sex at birth but identified as female.

### Study population, sample size, sampling procedure, and data collection techniques

A total of 20 in-depth interviews (IDIs), 6 focus group discussions (FGDs), and 10 key informant interviews (KIIs) were conducted. We recruited 60 transwomen to participate in the IDIs and FGDs (with 8 participants in each FGD). The total number of FGDs, IDIs, and KIIs was determined using the principle of theoretical saturation [35]. Data saturation was established using the approaches described by Guest, Namey [36] and Francis, Johnston [37]. This involved the identification of unique themes for base size and run length, which were subsequently used to calculate the saturation ratio. Base size was defined as the minimum number of interviews initially analysed to generate unique themes to be used in the denominator of the saturation ratio [36, 37]. A base size of 10 IDIs was established during the initial analyses, which provided the basis for progressive judgements regarding data saturation [37]. The run length was defined as the number of interviews within which we looked for, and calculated new information [36]. Operationally, a run referred to the number of consecutive interviews conducted with participants [36]. After the 10th interview, the stopping criterion described by Francis, Johnston [37] was applied until we obtained two consecutive interviews where no new information emerged from the interviews. Data saturation was determined using the theme on effect of GBV on access to HIV prevention services at healthcare facilities. All invited participants agreed to participate in the interviews.

KII were conducted with policymakers and individuals (including healthcare professionals, programme managers, peer leaders, and social workers) involved in the delivery of HIV-related prevention, care, and treatment services. The KII guide elicited information on their role in the delivery of HIV prevention, care, and treatment services, settings, forms, and perpetrators of GBV and how it affects access to and utilisation of HIV prevention, care, and treatment services among transwomen. For each KI, we asked how GBV independently affected access to and utilisation of HIV testing, disclosure of HIV status, enrolment and retention in care, and adherence to ART. We also asked about help-seeking following exposure to GBV, as reported in our earlier publication [16]. FGDs were used to obtain perspectives on GBV experiences and how they affect access to and utilisation of HIV prevention, care, and treatment services among transwomen communities. Respondents were asked about their understanding of GBV, perpetrators, and settings where transwomen experience GBV and how it affects the utilisation of services such as HIV testing, access to prevention measures such as pre- and post-exposure prophylaxis, and enrolment and retention in care for those living with HIV. FGDs were also used to recruit IDI participants because some experiences were sensitive, too personal, and emotional to be ethically shared in a group. The use of FGDs to recruit IDI and KI participants has also been reported in literature [38]. Therefore, IDIs were used to obtain detailed information on GBV lived experiences and how they affected access to and utilisation of HIV prevention, care, and treatment services. IDI participants were asked about their previous GBV lived experiences, including perpetrators, settings, disclosure of GBV experiences, sources of support, and how GBV affected their access to and utilisation of HIV prevention services. Data from KIIs, FGDs, and IDIs were triangulated to improve validity [39] and to obtain a comprehensive understanding of how GBV affects access to and utilisation of HIV prevention, care and treatment services. We piloted the IDI guide among 2 transwomen and the KI guide among 2 healthcare providers working with key populations in Mbarara City. This was aimed at improving clarity of the questions in the guide.

All FGDs and IDIs were conducted at the offices of key population organisations or key population healthcare facilities to ensure the privacy and safety of the participants. Snow balling and purposive sampling were used in the selection of respondents. On the one hand, we used partner organizations working with transwomen to identify individuals who could become primary respondents. The primary respondents then helped in the enrolment of secondary respondents. It is from the primary respondents that FGD and IDI participants were selected. The transwomen with lived GBV experiences of interest were identified during FGDs for interview as IDI participants. Key informants, however, were purposively selected based on their positions, experience, and presumed understanding of transgender persons, and gender-based violence and how it affects the uptake of HIV treatment and prevention services among transwomen. Key informants included policymakers, staff of civil society organizations working with transwomen, representatives of organizations providing access to justice services, and transgender-friendly sexual and reproductive health services providers. In order to ensure privacy and confidentiality, interviews were conducted in secluded and convenient places for the respondents. IDI, FGD, and KII guides were used to elicit data on GBV experiences and how it affects the uptake of HIV prevention, care, and treatment services.

### Data management and analysis

FGDs and KIIs were conducted by two researchers who included an interviewer and a note-taker. The role of the interviewer was to guide the discussions between the research team and the respondent/s while the note taker’s responsibility was to summarise interview proceedings and to observe. IDIs were conducted by only one interviewer. All interviews were recorded using the ICD-PX470 Sony digital voice recorder and later transcribed verbatim by experienced transcribers. We used transcribers who were conversant in both English and the language of the interviews (Luganda). These were also recruited on the basis of excellent computer skills and typing speed, attention to detail, and familiarity with the transwomen community. Transcribers were first requested to transcribe mock interviews that had been recorded by the investigators in order to assess their competencies. Upon completion of transcription, transcripts were read by two experienced researchers (MN and TS) several times, and codes and codebook definitions were developed based on the study objectives. Analysis involved reading the transcripts several times to identify and review themes that emerge from the data. We ensured reliability of the coding process by using two qualitative analysts. These were initially asked to independently code two transcripts and the extent of agreement between their coding schemes established. Disagreements were resolved through open discussion, as recommended by Chinh, Zade [40]. To make analysis easier, coding of the transcripts was aided by NVivo software. Our findings were validated through a stakeholder workshop, where policy makers, implementers and transwomen provided their feedback, which was also considered in the finalization of the manuscript. This was aimed at improving the methodological rigour of our study [41].

### Quality control and assurance measures

Research assistants with a minimum of a bachelor’s degree in public health, social sciences and any other related field were recruited. Furthermore, only individuals with a good command of English and the local dialect (Luganda) were recruited. Prior to data collection, research assistants were oriented through the study protocol, data collection tools, ethics and terminologies/slang used by transwomen. The data collection tools were translated from the original English version into Luganda by skilled translators. Pretesting of data collection tools was done to identify and correct any errors, and to allow the research assistants to familiarize themselves with the data collection tools.

## Results

### Background characteristics of the participants

The median age of the participants was 22 years, with the majority (76.7%) aged 24 years or younger. Nearly two-thirds (63.3%) of respondents had attained secondary level education. The majority (91.7%) had never been married. More than half of participants (60%) engaged in sex work as their main source of income (Table 1). Of the 10 KIIs, two were policy makers and 8 were directly involved in the delivery of HIV prevention, care and treatment services. Of the 8 KIIs involved in the delivery of HIV prevention, care and treatment services, 3 worked with a civil society key population-friendly healthcare facility, while the 3 worked with government owned key population-friendly healthcare facilities, 2 from civil society organisations working with transwomen.

**Table 1:** Sociodemographic characteristics of transwomen in the GKMA

Variable	Freq (N = 60)	Percentage (%)
District of residence		
Kampala	21	35.0
Mukono	6	10.0
Wakiso	33	55.0
**Age groups (years)**		
18–24	46	76.7
Above 24	14	23.3
**Education level**		
No formal education	1	1.7
Primary	10	16.7
Secondary	48	63.3
Tertiary	11	18.3
**Marital Status**		
Not married	55	91.7
Married	5	8.3
**People respondent lives with**		
Family home	14	23.3
Rented home without family members	21	35
Homeless	25	41.7
**Religion**		
Catholic	18	30
Protestant	14	23.3
Muslim	20	33.3
Pentecostal	5	8.3
Seventh-day Adventist	2	3.3
Other	1	1.8
**Main source of income**		
Salaried	9	15
Self-employed	6	10
Casual work	7	11.7
Sex work	36	60.0
Other sources	2	3.3
**Sex work (N = 36)**		
Street-based	2	5.6
Entertainment place-based	7	19.4
Residence/home-based	21	58.3
Other	6	16.7
**Length of time involved in sex work (n = 36)**		
From 1–5 years	30	83.3
From 6–10 years	4	11.1
From 11–15 years	2	5.6

### Effect of GBV on uptake of HIV prevention, treatment and care services

GBV negatively affected access to and utilisation of HIV prevention, care and treatment services at individual, community and healthcare setting levels. At individual level, emotional violence towards transwomen negatively affected disclosure of HIV status, ability to negotiate condom use, and adherence to ART. Sexual violence at individual level affected the transwomen’s ability to negotiate the use of condoms. At community level, both emotional and physical violence had an effect on travel to healthcare facilities. Emotional violence in healthcare settings made it difficult for transwomen to approach healthcare providers for HIV prevention, care and treatment services. Due to emotional violence, there was limited utilisation of HIV testing services and pre-exposure prophylaxis among transwomen.

There was also a delay in receiving appropriate healthcare and denial of healthcare services. Consequently, this made some transwomen shun healthcare facilities (Table 2).

**Table 2:** Effect of GBV on uptake of HIV prevention, treatment and care services among transwomen in the GKMA, Uganda

GBV experienced by transwomen	Effect of GBV on uptake of HIV prevention, treatment and care services		
	Individual level	Community level	Healthcare setting
Emotional violence: Inappropriate questioning, iciness, blackmail, inappropriate staring, gendered gossip, social disconnection, stigmatization	Inability to negotiate condom use Fear of disclosing HIV status and other health conditions to intimate partners and healthcare providers Non-adherence to ART	Fear to travel to healthcare facilities	Fear of approaching healthcare providers for services Limited use of pre-exposure prophylaxis Shunning health facilities Delay in receiving appropriate healthcare Denied healthcare services
Physical violence: Beating, partner fights, mob justice		Fear to travel to healthcare facilities	Shunning health facilities
Sexual violence: sexual assault, rape	Inability to negotiate condom use		

### Individual level

#### Inability to negotiate condom use

Nearly two-thirds of transwomen in our study engaged in sex work, although they also had intimate partners. Forced sexual intercourse by intimate partners, sex clients and employers limited transwomen’s ability to consistently use condoms which exposed them to the risk of HIV infection. Respondents pointed out that some of their intimate partners and employers forcefully denied their request to use condoms. Employers at times expected sexual favours in order ensure job security for transwomen. At times, some of the respondents were pushed or told to shut up when they resisted sex without condoms. In addition, forced sexual intercourse without condoms by sex clients and intimate partners was sometimes followed by physical violence.

“*I won’t say that it has never happened, having sex with someone and the condom is off. I didn’t realize the man had removed the condom and when I asked him about it, he held my head, pushed it back and told me to shut up. Then from that moment, should I call it rape?! It must have been rape because it didn’t feel consensual anymore. I was fighting to free myself and he didn’t want to use a condom.”* (IDI participant)

*“It may happen this way, you may be at work with your employer and you need to be promoted. Then he forces you to have unsafe sex and he refuses to use a condom. You will have nowhere to report and you will keep quiet in order to be promoted.”* (FGD participant)

*“You may be having a client or a partner and they tell you they are allergic to condoms. Even us transwomen we are at times not comfortable with condoms. At times they bring us cracks. It depends on the type.”* (FGD participant)

Respondents mentioned that when they are arrested sometimes, male police officers forced them to have sex with a promise of releasing them from the police cells. However, even after forcing them into unprotected sex without lubricants, transwomen are not released which hinders their access to test for HIV within the appropriate time.

*“Some male police officers will force you to have sex with them against your will. Some say that: ‘I first want to have sex with you and have a feel of how it is before I release you.’ They “use you” [have sexual intercourse] without lubricants or a condom and even after sexually abusing you, they end up not releasing you even when you need to test for HIV. You can’t report the abuse anywhere and you don’t even know their HIV status.”* (IDI participant)

Some reported being raped by inmate partners yet they could not report to police as they felt ashamed. This left them traumatized, and not aware of their HIV status.

*“Sometimes in prisons, when they arrest a transwoman like us, they may not know exactly your gender and some inmates just rape you. Sometimes you may fear to disclose it to the police officer that they sexually abused you. It’s shameful, sometimes you keep quiet but when you are traumatized*.” (FGD participant)

#### Fear of disclosing HIV status and other health conditions to intimate partners and healthcare providers

The respondents pointed out that insults and blackmail by their intimate partners hindered them from taking their pills (ART) at the right time and disclosing their HIV status. At times, some suffered from insults and blackmail on declaring their HIV status to their intimate partners. It was also revealed that some intimate partners disclosed the transwomen’s HIV status and the fact that their partners were on ART which in turn led to stigma. Due to the stigma arising from insults and blackmail initiated by intimate partners, some transwomen did not access healthcare facilities to seek HIV services due to fear of being known.

*“I would say you may fear the person [intimate partner] you stay with. Some spy on us [transwomen]. Yet, if you open up to them that you are HIV positive and that you are on ART medication, they may turn violent and may beat you up. He can start blackmailing you at your workplace and declare your HIV status. This can affect your utilisation of HIV services.”* (FGD participant)

*“When these people are in love, they share secrets and when they break up, the partners spill the secret and start publicly insulting each other. Some go ahead to declare how their partners are on ART. They [transwomen] keep on exposing themselves and this creates self-stigma among them to the extent that when they come around, they don’t want their friends to know what they have come to do at the healthcare facility.”* (Key informant)

*“If going out, I would go with him but he never allowed me to go out alone. I did not want to show him I was on ARVs. But I would take ARVs without his knowledge. I befriended the maids and the askari [security guard] who helped me link up with someone to sometimes deliver my ARVs whenever I needed them from the facility.”* (IDI participant)

*“I told my friend who was a straight girl, she went to the health center pretending to be the sick one and got me treatment. Though right now I separated from that friend of mine, so it is difficult to use her to get treatment. Since then, at times, I just buy medication if I have money.” (IDI participant)*

It was also noted that transwomen sometimes give false information to the health workers in fear of being judged. One of the KIs noted that transwomen do not reveal health conditions to health workers, especially when they have been raped, and the health workers at times offer treatment for a different issue when the real problem is concealed by the victim. Participants also revealed that they fear explaining issues related to their sexual organs because when they do, the health workers ask them a lot of questions and some of them judge them.

*“As for sexual violence, some of them get issues but when they come to the facility, they fear to open up that they were raped, they come up with another story for you to treat them. They face a lot of issues in bars.”* (Key informant)

#### Non-adherence to ART

The withdrawal of affection from close relatives and parents negatively affected adherence to ART among transwomen who were enrolled into HIV care. The withdrawal of affection meant that transwomen did not have people to confide in, which resulted in a feeling of loneliness and self-stigma which consequently discouraged them from taking ARVs leading to dropping out of care.

*“At times you feel lonely, I have no people I can confide in. I have my mother but she accepted me from a distance. She doesn’t want me to be with her. At times I feel like I don’t want to be with anyone. I feel self-stigma to the extent that I no longer want to take my ARVs. Sometimes you get tired of taking your daily ARVs when you have no father or mother you can lean on. They expected a lot from me but they feel disappointed.”* (IDI participant)

*“Yes, it [stigma] happens a lot, sometimes its self-stigma, sometimes its stigma from fellow patients. That is one reason why there are low retention rates among transwomen.”* (Key informant*)*

The shutting down of communication by intimate partners also played a key role in increasing transwomen’s non-adherence to ART and dropping out of HIV care. Some respondents noted that emotional abuse from intimate partners in the form of denying them a chance to communicate with friends, relatives and health workers made it difficult for them to access appropriate HIV prevention, care and treatment services. They expressed that this resulted in social disconnection, fear and depression.

*“We have experienced challenges with abusive partners. They deny us all our freedom to the extent that you don’t communicate with your friends and relatives, and they won’t let you access ARVs if you are sick. You can’t express yourself because you are like a prisoner. We at times stay with them because of the situation and they take care of us. Some victims end up being mentally affected, getting addicted to drugs, alcohol and becoming socially disconnected and experiencing fear and depression.”* (FGD participant).

### Community level

#### Fear to travel to healthcare facilities

Respondents revealed that, at times, they are afraid to travel to healthcare facilities or use certain routes in the community due to fear of or past experience of verbal abuse and physical violence such as being beaten up. Respondents mentioned that they are often insulted by community members and neighbours and stared at inappropriately because of the way they talk, walk and dress, which affects them emotionally, and discourages them from traveling to healthcare facilities.

*“Sometimes you may use some routes to the healthcare facility but people in that community may stare at you inappropriately. Then, there are those times when some community members undermine or even abuse us. It is an emotional torture and makes it uncomfortable for us to walk in public when going to seek HIV prevention, treatment and care services.”* (FGD participant, Kampala)

*“They violate us even on the way to the healthcare facilities. You may be moving then someone behind you starts to complain saying: ‘he is walking like a lady, the “bam-bam” (buttocks) is dancing left and right.’ So, you start wondering how you should walk so as not to attract any one’s attention.”* (FGD participant, Wakiso)

### Healthcare settings

#### Fear of approaching healthcare providers for services

Majority of the participants pointed out that fear of being laughed at by healthcare providers at general healthcare facilities and some key population clinics – which could in the long-run culminate into emotional violence – hindered uptake of HIV prevention, treatment and care services. As a result, some transwomen feared explaining to healthcare providers experiences of violence that would require HIV prevention, treatment and care services.

*“There are times we end up fighting, being raped or even having unprotected sexual intercourse. Yet, you cannot go and explain the details to the doctor because you fear that people at the healthcare facility will laugh at you.”* (IDI participant)

Majority of the participants pointed out that some healthcare providers asked inappropriate questions such as whether a transwoman had a wife or not, while others questioned their gender identity. Judgment, inappropriate questioning, and questioning of the transwomen’s gender identity played a key role in deterring some transwomen from approaching healthcare providers for HIV prevention, care and treatment services such as HIV testing, pre-exposure prophylaxis and ART initiation at some general and key population-friendly healthcare facilities.

*“First of all, I am a transwoman. I may wear my lipstick and in a weird way, the person (health worker) judges me. How will I start seeking HIV testing services? Yet when I reach the healthcare facility the healthcare provider will start lamenting that you look like a woman? The healthcare provider will start by asking me whether I have a wife or if am married. Because of such questions, I may find myself leaving the facility without taking an HIV test due to fear of declaring my gender identity. You will end up not knowing your HIV status because of the healthcare provider’s judgement of your gender identity.”* (IDI participant)

At many general healthcare facilities, healthcare providers resorted to preaching negatively about gender identity as opposed to offering HIV prevention, treatment and care services. Healthcare providers used the bible as a tool to emotionally taunt transwomen which eventually discouraged them from accessing HIV prevention, treatment and care services at general healthcare facilities. While preaching, some healthcare providers referred to transwomen as being stupid while others asked them to ‘leave’ their identity so as to be normal people.

*“Some doctors think they are very religious, so they at times preach to us instead of offering the services we need. Because we also have bibles on our phones, they don’t need to emphasize that God loves us. Since you know what may happen after reaching the healthcare facility, we would rather refrain and stay home.”* (FGD participant, Kampala)

*“Some healthcare providers blame them while in the treatment rooms. They keep asking them why they are transwomen. Other healthcare providers just preach to them to leave the transwomen identity so as to be normal people.”* (Key informant)

*“When you go to a private healthcare facility and express yourself to a doctor, he will start saying you are stupid. Why do you involve yourself in such behaviors (being a transwoman)? If they are born again, they will preach to you yet you have gone for a service. This will in the end divert your purpose of the visit.”* (IDI participant)

#### Limited utilisation of pre-exposure prophylaxis

While at healthcare facilities, patients and healthcare providers inappropriately stared at transwomen which limited them from uptake of pre-exposure prophylaxis. Some healthcare providers also deliberately referred transwomen to their colleagues, who they felt were more receptive to them. This consequently affected the utilisation of pre-exposure prophylaxis among transwomen.

*“When I go for PrEP [pre-exposure prophylaxis], people stare at me inappropriately. At times I feel bad and even lose focus. Sometimes, you may reach the hospital and the nurse looks at you and then she says: ‘we don’t understand you, go to nurse XX. She is the one who works on you people.’ Then other patients will start wondering why? Based on your appearance, other patients will start thinking that you are unique. The only issue is the way healthcare providers push us away in case you find one who doesn’t work on key populations. They at times shout at us so that even the person at a distance will wonder! This at times prevents me from going to the healthcare facility for PrEP.”* (IDI participant, Kampala)

#### Shunning healthcare facilities

Some transwomen shunned visiting general, private and some key population-friendly healthcare facilities due to fear of being judged by the patients and healthcare providers. Patients and healthcare providers stared and pinpointed them inappropriately. It was also reported that transwomen risked being beaten up at healthcare facilities due to their gender identity. As a result, some opted not to go to these healthcare facilities for HIV services.

*“Sometimes they (patients and healthcare providers) keep blaming and pinpointing us, hence making us feel stigmatized which makes us fail to come and access their medical services.”* (IDI participant)

*“GBV affects them in so many ways. As you know transwomen are different from the general population so when they come here, fellow patients look at them differently and this makes them uncomfortable.”* (Key informant)

*“We transwomen face GBV especially when we go to hospitals. When you are HIV positive, sometimes it’s hard when you present as a transwoman when people do not know you. If you are not safeguarded, you may be beaten up and dragged out of the hospital, and followed with insults like ‘are you a woman or a man?’”* (FGD participant)

The gendered gossip by some healthcare providers and binary patients which characterized many general healthcare facilities, and at times key population clinics, negatively affected enrolment into care, and the uptake of HIV testing, pre-exposure prophylaxis and antiretroviral therapy. The gendered gossip often resulted in a breach of confidentiality of transwomen’s health status and gender identity.

*“There was a time I visited one of the healthcare facilities. I had gone for medical services because I wasn’t feeling well, and I was in fear. I was served well but after exiting the doctor’s room, he shared my condition with someone who later told all community members in my circles what I was suffering from. I felt bad, I hated that facility and I never went back. It is now two years down the road!”* (IDI participant)

*“Facility YYY is an LGBT healthcare facility but sometimes I cannot access it because it also serves the general community. When I get there, other patients begin to gossip about my identity. The gossip makes it uncomfortable to access the healthcare facility.”* (IDI participant)

*“There is a doctor in a certain hospital that gave me an injection. He first asked me how I identify. I told him that I am a female and he said I was lying. When we went to the injection room, he gave me an injection and my hand got swollen. That injection was about to take my life! When I confronted him, he told me he wasn’t aware of transwomen and that he thought I was a person who was pretending or maybe I wasn’t normal. It affected me to the extent that didn’t want to go back to that hospital. I had it in mind that if I go back, I might face the same doctor.”* (IDI participant)

#### Delay to receive appropriate healthcare

A few respondents mentioned that they did not receive timely healthcare due to their gender identity. The study showed it was a common practice for healthcare providers, especially those working in general and public healthcare facilities, to keep asking transwomen how they benefited from being transwomen instead of cisgender. This led to delays in providing transwomen with appropriate care.

*“Yes, we still have nurses who haven’t embraced who we are, but we just bear with them especially in public healthcare facility YY. The nurses keep asking how we benefit as transwomen, and at times you can send in a client (transwoman) and in the absence of a peer the nurses take their time (take long) to work on them.”* (FGD participant)

*“They say that life comes first but when you go there (to the healthcare facility), instead of working on you, some healthcare providers first ask you nonsense or call their friends: ‘XX, XX [name of healthcare provider], come and see them. Oh my God, XX, here are the ones who do them [transwomen].’ If it were you, would you go back for any healthcare service?* (IDI participant)

#### Denied healthcare services

Some respondents reported being denied healthcare services as a result of providers’ transphobia. Healthcare providers in some general and key population-friendly healthcare facilities exhibited displeasure toward transwomen to the extent of refusing to provide healthcare services to them. In some instances, the healthcare providers referred transwomen to key population-friendly healthcare facilities, or healthcare providers who they felt were trans-friendly and accommodative.

*“You can enter a hospital that is not even aware of transgender persons. Then they ask: ‘who are you? How come you behave like a woman?’ A nurse who is attending to you ends up calling other nurses saying: ‘come and see this “thing” [transwoman], where does “it” even come from?’ Instead of attending to you, they start talking about you and they may even end up not working on you. They may tell you: ‘we don’t have this medicine, try another facility.’ Yet, they have the medicine but they just don’t want to work on you.”* (IDI participant)

*“Sometimes transwomen are denied treatment by some healthcare workers and are referred to other key population-friendly healthcare facilities. We are also blamed even when we come for treatment. This discrimination leaves us stigmatized.”* (FGD participant)

*“Some healthcare providers blame them while in the treatment rooms. They keep asking them why they are trans women. Some healthcare providers even refuse to treat them and refer them to those healthcare providers they know to be key population-friendly. Other healthcare providers just preach to them to leave the transwomen identity so as to be normal people.”* (Key informant)

## Discussion

The current study explored how GBV affected access to and utilisation of HIV prevention, care and treatment services. Emotional violence suffered at individual and community levels, and in healthcare settings, was the main hindrance to the utilisation of HIV prevention, care and treatment services. At individual level, it resulted in a fear of disclosing HIV status and other health conditions to intimate partners and healthcare providers, non-adherence to ART and inability to negotiate condom use. At community level, it created fear that made it difficult for transwomen to approach healthcare providers for services, led to limited use of post-exposure prophylaxis and HIV testing services, delays in receiving care, and at times being denied healthcare services. Physical violence, such as fights with a partner and beatings in the community and healthcare settings, resulted in fear of traveling to healthcare facilities, while sexual violence such as rape compromised the ability to negotiate condom use.

This study also revealed that gender-based violence compromised the ability of transwomen to decide to seek HIV prevention, care and treatment services. Emotional violence, mainly exhibited through insults and blackmail and shutting down communication by intimate partners hindered access to and utilisation of HIV prevention, care and treatment services as some intimate partners deliberately disclosed their partners’ HIV status, which consequently affected adherence to ART. Shutting down communication between transwomen and their friends, relatives and health workers negatively affected uptake of healthcare services which is concerning because friends and relatives play an important role in HIV prevention, care and treatment. They usually play a critical role in reminding individuals living with HIV to take their ART medication, and provide financial, emotional and social support. This kind of support is widely reported as vital in ensuring access to and utilisation of HIV prevention, care and treatment services, and associated outcomes.

Sexual violence in the form of rape compromised the ability of transwomen to negotiate the use of condoms. This study revealed that transwomen were often unable to use condoms since they were forced into sexual intercourse by their employers, intimate partners, security personnel and sex clients. The fact that they were not able to negotiate condoms use should be worrying due to the known risks of unprotected sexual intercourse. Failure to develop and implement strategies/ interventions targeting a reduction in sexual violence, particularly rape, is likely to contribute to an increase in the HIV prevalence among this sub-group and the people with whom they have sex or are forced to have sex. Sexual violence and its effect on access to and utilisation of HIV services is widely documented.

The study revealed that the lack of acceptance of the transwomen by fellow patients at healthcare facilities due to their gender identity impeded their ability to seek HIV prevention, care and treatment services. This manifested itself through beating the transwomen, dragging them out of the healthcare facilities, and inappropriately staring and gossiping, which affected their self-confidence and ability to seek the necessary HIV services. Some transwomen avoided such facilities altogether in anticipation of experiencing emotional and physical abuse while at the facility. These findings corroborate those reported in a qualitative study conducted by Lanham, Ridgeway [42] in Latin America and the Caribbean that indicated inability to seek care among transwomen as a result of the emotional violence perpetrated by fellow patients at healthcare facilities. Ssekamatte, Isunju [12] attributed this violence to the fact that transwomen don’t behaviourally conform to expected gender roles and norms and are therefore prone to transphobia from fellow patients and healthcare providers. Our study highlights the importance of stigma- and discrimination-free healthcare settings when using HIV/AIDs related prevention, treatment and care services.

Our study also indicated that the lack of acceptance of the transwomen by some healthcare providers – for example, preaching and gossiping negatively about their gender identity and HIV status – compromised their ability to seek HIV prevention, treatment and care services. Respondents noted that they refrained from seeking services at healthcare facilities because some healthcare providers referred to them as stupid while others asked them to “leave” their identity and become “normal” people. In addition, transwomen in this study reported breaches of confidentiality about their HIV status and gender identity by some healthcare providers. This emotional abuse discouraged them from not only approaching the healthcare workers, but also trusting them and disclosing vital information required to arrive at proper diagnosis and treatment. These findings reaffirm those of our earlier study which reported breach of confidentiality in healthcare settings as a barrier to access and utilisation of HIV-related services among transwomen [12]. These findings could be attributed to inadequate training of healthcare providers on sexual orientation and gender identity especially in relation to transwomen, their unique healthcare needs and different forms of GBV, especially those perpetrated within healthcare settings. The findings could also be attributed to the deeply rooted cultural belief about gender that cannot be easily erased except with continued dialogues and awareness sessions about the trans gender identity.

Results indicated that GBV is also linked to unfriendly healthcare facilities. Respondents anticipated being laughed at, being asked inappropriate questions about their gender identity, and abused by healthcare providers if they expressed their healthcare needs, issues and experiences of GBV. This compromised their ability to approach healthcare providers and freely express their issues, which could have blocked their opportunity to seek HIV prevention, care and treatment services in real time. The study also reported instances of deliberate refusal to treat some transwomen where the healthcare providers were condemning them for behaving like girls which later forced them to flee such healthcare facilities. Similarly, a review of existing literature showed discrimination in healthcare settings exhibited through denial of care [1, 12, 43, 44]. Such emotional abuse and discrimination in healthcare settings distresses transwomen, ignites self-stigma and affects their self-confidence, which impedes their desire and ability to access appropriate care. In addition, transwomen may lose trust not only in the service providers but also in the services provided leading to a delay in seeking HIV prevention and treatment services [1]. There is therefore a need to train healthcare providers in key population healthcare facilities on both intentional and unintentional discriminatory behaviors that are likely to impact on transwomen’s uptake of HIV-related services.

At community level, transwomen reported that they were unable to reach/travel to healthcare facilities due to fear of community-perpetrated emotional abuse, exhibited through verbal abuse and inappropriate staring at them. This was because the transwomen’s behavior and dress code defied the societal and gender norms. As a result of this abuse, transwomen were prone to emotional distress, dissociation and social exclusion, which affected their access to healthcare services. These findings concur with those reported in a study conducted among transwomen sex workers in Uganda [12]. Communities should therefore be sensitized on transgender identity and different forms of GBV, including non-physical forms such as emotional violence. This will create an enabling environment for transwomen to travel to healthcare facilities for HIV-related services and any other healthcare services needed.

### Strengths and limitations of the study

This study demonstrates the lived experiences of transwomen who have suffered the effects of GBV on uptake of HIV prevention, treatment and care services. Besides the effects of physical violence, the study brings to light the effects of emotional and psychological forms of violence which inhibit uptake of HIV prevention, treatment and care services. The names of healthcare facilities have been withheld for ethical concerns. The findings are however limited in terms of geographical space and some of the findings may not be generalizable due to contextual variations.

## Conclusions

It is noted that emotional violence is the most widespread form of GBV at individual and community level and, worse, in healthcare settings and that this precipitates fear of disclosing HIV status and other health conditions to intimate partners and healthcare providers, inability to negotiate condom use, and non-adherence to ART at individual level. At community level, both emotional and physical violence instil fear to travel to healthcare facilities. Emotional violence in healthcare settings has a number of effects, including fear of approaching healthcare providers for services, limited take-up of HIV testing and pre-exposure prophylaxis services, delays in receiving appropriate healthcare, being denied healthcare services and shunning healthcare facilities. There is a need to develop and implement strategies/ interventions targeting a reduction in GBV, given its effect on the transmission of HIV. Interventions should include strategies to sensitize communities at accepting transwomen. Healthcare providers should create an environment at the healthcare facilities where transwomen are able to approach any healthcare provider of their choice so as to increase uptake of HIV prevention, treatment and care services among transwomen. Specifically, there is a need to train healthcare providers on gender and sexual diversity and the unique health needs of transwomen. The use of escorted referrals can also mitigate the effects of GBV on access and utilisation of HIV prevention, care and treatment services.

## Data Availability

The data used for this manuscript is available from the corresponding author on reasonable request.

## Abbreviations

GBV: Gender-based Violence
GKMA: Greater Kampala Metropolitan Area
HIV: Human Immunodeficiency Virus
STIs: Sexually Transmitted Infections
